# Do they speak like me? Exploring how perceptions of linguistic difference may influence patient perceptions of healthcare providers

**DOI:** 10.1080/10872981.2022.2107470

**Published:** 2022-08-01

**Authors:** Donghee N. Lee, Myiah J. Hutchens, Thomas J. George, Danyell Wilson-Howard, Eric J. Cooks, Janice L. Krieger

**Affiliations:** a Department of Population and Quantitative Health Sciences, Division of Preventive and Behavioral Medicine, UMass Chan Medical School, Worcester, USA; b Department of Public Relations, College of Journalism and Communications, University of Florida, Gainesville, USA; c Department of Medicine, College of Medicine, University of Florida and University of Florida Health Cancer Center, Gainesville, USA; d Department of Natural Sciences, Bethune-Cookman University, Daytona Beach, USA; e STEM Translational Communication Center, University of Florida and University of Florida Health Cancer Center, Gainesville, USA; f Department of Advertising, College of Journalism and Communications, Health Outcomes and Biomedical Informatics, College of Medicine, University of Florida, Gainesville, USA

**Keywords:** Linguistic biases, African American english, diversity in healthcare, communication inequities, racial stereotypes, virtual health clinicians, diversity and equity

## Abstract

The increased utilization of telehealth has provided patients with the opportunity to interact with racially diverse healthcare providers (HCPs). While evidence of racial stereotypes in healthcare is well documented, less is known about whether linguistic cues increase or decrease racial bias in healthcare interactions. The purpose of this pilot study was to use virtual clinicians (VCs) to examine how varying linguistic features affect patient perceptions of Black-identifying HCPs. Participants (*N* = 282) were recruited to participate in an online pilot study using a two-arm posttest-only experimental design. Participants were randomly assigned to interact with a Black VC that used vocal cues associated with either Standard American English (SAE) or African American English (AAE) on the topic of colorectal cancer. After the interaction, participants completed a posttest questionnaire. Resulting data were analyzed using mediation.

## Introduction

People regularly make attributions about others based on the way they speak, such as the geographic region of origin and socioeconomic status [1,2]. In the USA, a person with ‘country talk’ would likely be assumed to originate from a Southern state[3]. In healthcare, patients’ perceptual biases with certain speech styles (i.e., linguistic cues) can influence the assessment of their healthcare providers (HCPs). Understanding the role of linguistic biases in healthcare can help combat racial stereotypes that contribute to the vast underrepresentation of Black-identifying HCPs[4]. While Black-identifying Americans consist of 13.4% of the total U.S. population[5], they are vastly underrepresented in the clinical workforce. Only 5% of active physicians [6], 3% of practicing oncologists[7], 3.6% of medical school faculty[8], and 3.9% of oncology fellows [9] self-identify as Black Americans. Representation is even more disparate for other cancer specialties, including surgical [4] and radiation oncology[10]. The American Society of Clinical Oncology has made several efforts to diversify the healthcare workforce, including prioritization of health equity research, mentorship programs, and the launch of the Oncology Talent and Diversity Program to foster the pipeline of racially underrepresented medical trainees[4]. While these programs signal an increased focus on equity, research about the role of linguistic cues and biases on perceptions toward Black-identifying HCPs may elevate understanding of challenges associated with recruiting and retaining Black-identifying HCPs in the field.

According to Communication Accommodation Theory[11], perceived similarity with others influences how individuals construct and interpret messages [12,13]. In a multiracial setting, as in the USA, linguistic cues often signal a certain racial or ethnic group membership, which can influence perceptions of non-Black-identifying patients toward Black-identifying HCPs. According to the linguistics literature, African American English (AAE) represents a speech style spoken by individuals who identify as Black Americans in the USA [14,15]. Conversely, Standard American English (SAE) represents a variety of English lacking distinct regional, racial/ethnic, or socioeconomic attributes [16,17]. Historically, AAE speakers have experienced discrimination and prejudice[18], as the dominant society has framed AAE with incompetence, illiteracy, and ‘street slang.’[19] AAE speakers, who typically identify as Black Americans, have experienced discrimination because of the negative linguistic biases associated with AAE [20–22]. This form of ‘raciolinguistic exceptionalism’ where performance is judged through the lens of racist views on language acts to perpetuate racist structural narratives on the ‘Black’ social identity[23].As such, understanding perceptual biases toward AAE may explain the challenges of Black-identifying HCPs or medical trainees during their patient encounters and ways to mitigate these psychosocial factors potentially contributing to the underrepresentation of Black-identifying HCPs.

Perceived similarities and differences are an important predictor for the evaluation of HCPs [24–27]. Conversely, perceived dissimilarity has been associated with biases, prejudices, and negative evaluations against the person and interactions [28,29]. In multiracial healthcare settings, perceived racial similarities often predict greater patient satisfaction [30]. It is well documented that humans tend to accommodate, or adapt, to another person’s speaking style to increase perceived similarity. For instance, it has been observed that some Black-identifying HCPs may speak with AAE to build rapport and provide a comfortable environment for Black-identifying patients [27]. In addition, Black-identifying HCPs who have linguistic competence in both SAE and AAE ‘code-switch’ between SAE when speaking with their colleagues and AAE with Black-identifying patients [31]. While it is clear that Black-identifying HCPs make linguistic accommodations in the clinical environment, there has not been a clear test of the relationship between speech styles and patient evaluations of their HCP. Demonstrating how individuals respond to different linguistic cues can provide insight into the lived experience of Black-identifying HCPs by demonstrating whether there are differences in how patients react to different speech styles. In this study, we take a raciolinguistic perspective to understand how the society interprets the linguistic patterns of marginalized populations, specifically as it affects the Black-identifying population [32,33].

Outside the context of race and language, researchers have tested the effect of perceived similarity between patients and HCPs using virtual clinicians (VC) [25,26,34]. For instance, a clinical trial involving virtual nurses found that patients reported increased satisfaction, trust, liking, preference, perceptions of caring, and willingness to work with the nurses that they personally identified with[26]. Relatedly, an mHealth intervention study involving Black men living with HIVs revealed that participants found it more acceptable to interact with an embodied VC that was customized to match their demographic characteristics (race, gender) and shared experiences of living with a stigmatized illness[24]. Thus, patients’ perceived similarity and dissimilarity with Black-identifying HCPs may influence their evaluation of Black-identifying HCPs. Manipulating speech styles in a telehealth intervention using a VC is one way to test the effect of patients’ perceptions of linguistic similarity with a VC on their evaluation of the VC.

The purpose of this pilot study was to determine whether individuals’ identification with the VC’s speech styles influenced their perceptions toward the VC. Despite the variety of speech spoken by self-identified Black individuals (e.g., Creole), we focus on AAE-speaking Black-identifying HCPs in this study because of the documented evidence against AAE. By using a sample of the general population, we examined how linguistic biases and perceptions toward AAE influenced evaluations of a Black VC. We hypothesized that participants (a general sample of population, mostly Non-Hispanic White), who do not associate with AAE, would be less likely to identify with AAE-speaking Black VC, and there would be a negative association between AAE and participants’ evaluation of the AAE-speaking VC, mediated by their low perceived linguistic similarity.

## Methods

### Sample and design

In April 2021, we recruited a nationally representative convenience sample of multiracial/ethnic participants (N = 282) from Amazon Mechanical Turk (mturk.com) to conduct a two-arm randomized experiment. Participants were eligible if they were 18 years or older and resided in the USA. Potential participants reviewed a brief study description on MTurk. If they were eligible, participants were directed to a Qualtrics survey and asked to provide consent. Then, they were randomized to watch one of two videos of a Black male virtual clinician (VC) speaking in either Standard American English (SAE) or African American English (AAE). The video was adapted from a larger study examining the efficacy of a culturally targeted mHealth intervention promoting cancer screening [25,34–36]. Random assignment occurred by using randomization flow in Qualtrics. This computer-generated algorithm allowed us to ensure an even distribution of our samples into each condition. After viewing the video, participants completed measures on perceived linguistic similarity and perceptions of the VC. After study completion, participants were thanked and compensated. All procedures were approved by the University of Florida’s Institutional Review Board.

### Measures

Demographics. Participants reported their age (treated as continuous), gender (male and female), race (collapsed to White/Caucasian and others), ethnicity (Non-Hispanic/Latino and Hispanic/Latino), and education (collapsed to <some college, >college graduates). Demographic data were collapsed to create dichotomous variables required for analyses.

Linguistic cue manipulation check. Manipulation of linguistic cue was checked using one item: ‘How likely is that the virtual agent speaks like White/Caucasian’ to assess participant’s perceived race/ethnicity of the Black VC[37]. Scores were on a scale of 1 (Extremely likely) to 5 (Extremely unlikely).

Perceived linguistic similarity. Perceived linguistic similarity was assessed by using two items from an adapted homophily scale: ‘Does not speak like me – Speaks like me’ and “Speaks differently from me – ‘Speaks similarly to me.’[38] Scores were on a scale from 1 to 7.

Source evaluation. Source (Black VC) evaluation was assessed by using 14 items adapted from trait evaluation and negativity scale (e.g., Intelligent, friendly, judgmental, and ungrateful) [28]. Scores were on a scale from 1 (Not at all) to 7 (Very much).

### Statistical Analysis

All analyses were conducted using SPSS v. [28]. An independent t-test was performed to check manipulation. Descriptive statistics were used to calculate distributions of speech styles, perceived linguistic similarity, and source evaluation. First, we ran bivariate correlations between speech styles and the two outcome variables: perceived linguistic similarity and source evaluation. Speech styles and perceived linguistic similarity were significantly correlated, but source evaluation was not. Then, we ran a bivariate correlation between perceived linguistic similarity and source evaluation, and they were significantly correlated. We then used Hayes PROCESS Macro v.4.0 to estimate the mediation of perceived linguistic similarity on the association between speech style and source evaluation. An alpha 0.05 was used to identify statistical significance.

## Results

### Participants

On average, participants were 36.4 years old (SD = 11.04), identified mostly as White (67.4%) and non-Hispanic (72.3%), male (70.2%), and college graduates or above (77.3%). See Table 1.

**Table 1.** Sample characteristics, N = 282^a^.

	N (%) or mean (SD)
Age (years)	36.4 (11.04)
Race	
White	184 (65.2%)
Others^#^	98 (34.8%)
Ethnicity	
Hispanic or Latino	78 (27.7%)
Not Hispanic or Latino	204 (72.3%)
Education	
Some college or below	64 (22.7%)
College graduates and above	218 (77.3%)
Gender	
Male	198 (70.2%)
Female	84 (29.8%)

a Participants were recruited from the online crowdsourcing platform, MTurk.

# Others include Black or African American (n = 20; 7.1%), Asian (n = 56; 19.9%), American Indian, Alaska Native, Native Hawaiian, or other Pacific Islander (n = 12; n = 4.3%), Other (n = 1; 0.4%), or multiple races (n = 9; n = 3.2%).

### Manipulation check

The perceived difference between AAE and SAE was statistically significant, t(280) = −2.22, p = .03, 95% CI [−.47, −.03]. Participants in the SAE condition perceived the VC speaking in SAE to sound like White/Caucasian (M = 2.75, SD = .93) more than the VC in the AAE condition (M = 2.50, SD = .94).

### Descriptive statistics

Perceived linguistic similarity items were summed and averaged (M = 5.56, SD = 1.25, SBr = .71). Higher scores indicated higher perceived linguistic similarity. Source evaluation items were summed and averaged. Six items were reverse coded (M = 4.38, SD = .82, α = .89). Higher scores indicated more positive evaluation of the source.

### Regressions examining associations between speech styles and outcomes

The association between speech style and perceived linguistic similarity was statistically significant, with the participants reporting lower perceived similarity with AAE, b = −.35, SE = .15, p = .02, 95% CI [−.64, −.05]. The association between speech style and source evaluation was not statistically significant, b = −.06, SE = .10, p = .53, 95% CI [−.25, .13].

### Mediated association of speech styles and source evaluation

**Figure 1.**
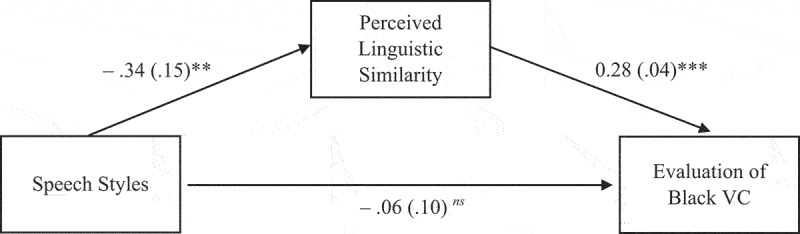
Indirect pathway from speech styles to VC’s evaluation via perceived linguistic similarity (N = 282)^a^.

The indirect effect of speech style on the source evaluation was mediated by participants’ perceived linguistic similarity, b = −.10, SE = .04, 95% CI [−.18, −.02]. The association of speech style and perceived linguistic similarity was statistically significant, b = −.34, SE = .15, p = .02, 95% CI [−.64, −.05]. The association between perceived linguistic similarity and source evaluation was statistically significant, b = .28, SE = .04, p < .001, 95% CI [.21, .35]. See Figure 1.

**p < 0.01, ***p < 0.001, ns: not statistically significant. ^a^N = 282 participants were randomized to view one of the two speech style conditions (Standard American English (SAE) and African American English (AAE)). Hayes PROCESS Macro was used to model the mediated association between speech type and outcome variables. The SAE condition was the reference group. AAE (vs. SAE) was associated with decreased perceived linguistic similarity. Decrease in perceived linguistic similarity was positively associated with decreased evaluation of Black VC. The unstandardized coefficient and the standard error denote each path. Covariates were excluded from the analysis because the study used random assignments.

## Discussion

Overall, our study is among the first to use a telehealth delivery model to examine the effect of linguistic cues on perceptions of Black-identifying HCPs. Our results show that participants were less likely to positively evaluate the AAE-speaking Black VC than the SAE-speaking VC, and this association was mediated by their low perceived linguistic similarity with the VC. However, it is important to note that the direct effect of AAE on the evaluation of VC was not significant. This is notable because it emphasizes the importance of linguistic similarity as the mechanism through which speech style influences liking. The insights gained by our work advance understanding of the linguistic biases associated with AAE speakers in the medical context.20 – [22,39] We found that linguistically dissimilar audiences demonstrate the greatest bias towards speakers of AAE in clinics. Overall, our findings suggest that linguistic biases may contribute to communication inequities and discrimination against HCPs who speak differently from specific patient populations, which may adversely affect HCPs of minoritized racial/ethnic groups.

Additionally, our work provides preliminary evidence to consider implicit linguistic biases when developing medical education and training curriculum to promote diversity and inclusion. In our sample, the associations of Black VC’s speech styles and evaluation of the VC were mediated by participants’ perceived linguistic similarity with the VC. Participants were significantly less likely to identify with AAE compared to SAE, which was associated with poorer evaluation of the AAE-speaking VCs. Outgroup negativity bias [28,29,40] potentially explains this association. People tend to show a lower liking of linguistically dissimilar others [11,12]. Thus, given the demographics of our sample, our participants were likely to perceive AAE as a cue for a dissimilar social group membership. Our finding reveals the importance of linguistic variation as an important contributor to social inequities in medicine, as it may explain why racial/ethnic minority individuals are expected to adopt SAE for acceptance and successful integration into the mainstream medical culture, which individuals self-identifying as Black Americans often experience[41]. This practice may perpetuate raciolinguistic exceptionalism through structural oppression of the marginalized linguistic styles[23].

Our findings contribute to a body of literature documenting bias toward Black-identifying HCPs and provide a potential mechanism for better understanding that bias. Prior work found that Black-identifying providers are more likely to receive lower patient satisfaction scores than White providers[42], which can adversely affect their hiring process. Indeed, hiring discrimination against speakers of AAE has been shown in other areas[22]. In our study, participants were more likely to negatively perceive AAE-speakers. Because most of the participants in our sample identifying as White and Caucasian, they were less likely to speak AAE than those who self-identify as Black-identifying patients. This indicates ingroup bias associated with language[11], explaining why White participants perceived AAE negatively. Future studies should examine whether perceptions of AAE may differ between Black-identifying patients and White patients, and which speech style is more preferred by different racial groups. Although non-Black-identifying individuals do not respond with explicit negativity toward AAE, they are more likely to critically evaluate AAE-speakers when the topic is abstract and intellectual[39]. Furthermore, negative and relatively automatic reactions to non-normative accents and speech have been documented in the previous research[43]. It is likely that such biases have influenced Black-identifying HCPs to code-switch to SAE to accommodate to the linguistic needs of the non-AAE speaking colleagues and patients. Such practice may reinforce the cultural normativity and endorsement of SAE during professional encounters, and potentially underaccommodate to Black-identifying patients’ communication needs. This non-accommodation has been translated into inequities in healthcare practices. For instance, racially dissimilar (e.g., Non-Hispanic White) physicians who are likely experiencing communication through an intergroup (interracial) lens may not recognize situations requiring communication accommodation (e.g., exercising cultural sensitivity during both listening and speaking), which has led to labeling Black-identifying patients as uncooperative [44,45]. Our findings suggest that such implicit biases towards AAE persist to this day and such negative perceptions toward AAE translate into poor evaluation of AAE-speaking HCPs.

In order to truly diversify the workforce, medical education and training programs should aim to change the healthcare culture in ways that embrace and promote diverse cultures in all areas, including communication practices. In some cases, Black-identifying physicians may possess the linguistic flexibility to engage in code-switching between SAE and AAE [18,27,41]. This cultural fluency provides a significant strength to the healthcare organizations to meet the needs of diverse patients. Taking a raciolinguistic perspective [32,33], the findings of this work also imply the need for multi-level efforts to reassess institutional cultures that produce race-based evaluations of language deficiency.

Medical education programs should recognize the importance of linguistic vitality to increase diversity among HCPs and patients. Our finding can also benefit non-Black-identifying HCPs so that they can better align their intention with practice, such that they are mindful of their possible implicit biases and effortfully accommodate to culturally diverse patients. Taken together, the findings of this study provide critical insight that should be considered when developing a medical training tool aimed at promoting diversity and inclusion among medical experts and trainees. More work is needed to diffuse racial stereotypes and representations to promote communication equity in healthcare.

This study has several limitations. First, we did not measure implicit biases associated with AAE or SAE. However, our work was informed by the extensive evidence on the negative stereotypes and biases associated with AAE [19–22,46]. In addition, participants were randomly assigned to the condition, making it unlikely to influence study results. Future studies may adopt implicit association test [47] to further examine how linguistic biases influence perceptions of AAE-speaking Black VCs. Secondly, this study only used a male Black VC. While this enabled us to ensure that VC gender was not confounded, we acknowledge that there are different societal expectations toward male and female behaviors (e.g., female HCPs exhibiting greater empathy than male HCPs)[48]. Thus, future studies may examine possible interaction effects between gender and race on perceptions toward HCPs. Additionally, the current study was a pilot study and as such used convenience sampling. While MTurk has been found to be representative for social phenomena[49], its effect may not generalize to health behaviors[50]. Given that MTurk samples have higher education than the general population, we would expect to see stronger effects among the general population. In this study, we decided to use crowdsourced samples because of ethical considerations that would make this experiment difficult to perform in practice. The next phase of this project will entail using probability samples to improve generalizability involving a sample of Black-identifying participants speaking in AAE to provide AAE-speakers’ perspectives on Black VC’s speech styles. Additionally, the next phase of the study will compare the differences between AAE-speakers and non-AAE speakers by purposefully oversampling from each group. Further, future research can be compared across different demographic groups (e.g., race, ethnicity, age, and education) to examine their perceptions of SAE and AAE. Finally, findings from our study will likely apply to AAE-speaking Black-identifying HCPs, who are likely to be born in the USA. However, future research should explore the difference between perceptions of AAE and other speech variations and accents, especially the nuances of linguistic prestige involving different accents. For example, a Black-identifying HCP with a British accent may be viewed differently from Black-identifying HCP speaking in AAE, due to higher social prestige associated with the former [51,52].

## Conclusions

Linguistic stereotypes influence social interactions, including those in the medical context [11,12]. Sadly, stereotypes of AAE reflect the history of racism in the USA [19–21]. Often, efforts to diversify the workforce focus exclusively on self-reported race and ethnicity. However, the strong relationship between language and culture indicates that linguistic diversity is essential to improving health organizations. To meet this challenge, we do not suggest training minority physicians to codify their speech in a more accepted pattern determined by those in power. Instead, we suggest to further explore how race and language interplay, and the role of systems that influence the conceptualization and interplay of race and language [33]. This paper contributes a first look at the relationship between linguistic stereotyping and evaluation of HCPs in the virtual environment. The data suggest that people who do not speak AAE report reduced linguistic similarity and lower liking when interacting with a Black VC that uses a speech style with the phonological and morphosyntactic features of AAE as compared to a Black VC speaking SAE. The strong association between speech similarity and liking of the VCs suggests that speakers of AAE would rate AAE-speaking VCs positively. This is an important area for future research to pursue. While we cannot generalize these results in face-to-face interactions, it indicates the importance of further research on linguistic biases on outcomes in healthcare organizations. Often implicit, linguistic bias may go undetected and negatively influence efforts to enhance the diversity of our clinical workforce. These types of experiments are often not feasible to conduct in clinical settings. Future research should explore how VC platforms can inform understanding of the role of speech on healthcare outcomes.

## Data Availability

Data are available upon request.
